# Supplementary material for: Photoimmunotherapy Using Cationic and Anionic Photosensitizer-Antibody Conjugates against HIV Env-Expressing Cells
Source: Int J Mol Sci. 2020 Dec 1;21(23):9151. doi: 10.3390/ijms21239151 (PMC7730620; doi:10.3390/ijms21239151)
Supplement: Supplementary file 1 [file ijms-21-09151-s001.zip › Supplementary materials with videos 1 and 2/Supplementary materials.pdf]

SUPPLEMENTARY INFORMATION

Photoimmunotherapy using cationic and anionic photosensitizer-antibody conjugates against HIV Env-expressing cells

Mohammad Sadraeian[1], Calise Bahou[2], Edgar Ferreira da Cruz[3], Luiz Mário Ramos Janini[3], Ricardo Sobhie Diaz*[3], Ross W. Boyle*[4], Vijay Chudasama*[2], Francisco E. G. Guimarães*[1]

[1] Instituto de Física de São Carlos, Universidade de São Paulo, Caixa Postal 369, São Carlos, SP, CEP 13560-970, Brazil

[2] Department of Chemistry, University College London, London WC1H 0AJ, UK

[3] Laboratório de Retrovirologia, Universidade Federal de São Paulo, São Paulo, Brazil

[4] Department of Chemistry, University of Hull, Cottingham Road, Hull, HU6 7RX, UK

*** Corresponding authors:**

Francisco Eduardo Gontijo Guimarães, email: guimaraes@ifsc.usp.br, +55(16)33739792

Vijay Chudasama, email: v.chudasama@ucl.ac.uk

Ross W. Boyle, email: r.w.boyle@hull.ac.uk

Ricardo Sobhie Diaz, email: rsdiaz@catg.com.br

Word count: Main text 760

Supplementary Results

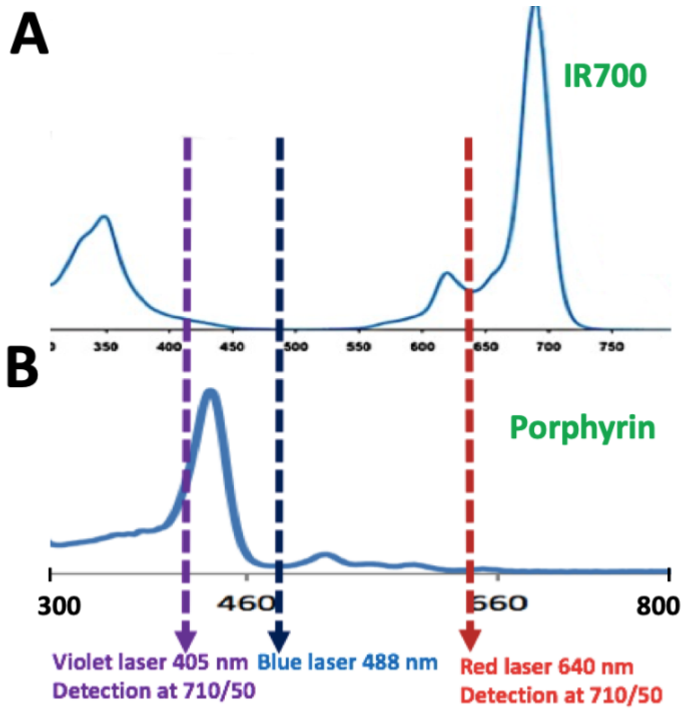

C

Flow cytometer Laser Excitation (nm)	Single Excitation (nm)	Intensity of Emission at 725 nm (a. u.)
Violet	405	1.163
	432	3.902
	433	3.779
Blue	488	0.165
Yellow /Green	561	0.352
Red	640	0.068

Supplemental Figure S1. Analysis of Excitation and emission spectra before flow cytometry and microscopy studies. **(A and B)**. Flow cytometry BD LSR Fortessa equipped by five lasers, including UV laser 370 nm, violet laser 405 nm, Blue laser 488 nm, yellow/green laser 561 nm and red laser 640 nm. Theoretical analysis of UV-Vis spectra showed blue laser 488 nm is the best laser for irradiation of secondary antibody-FITC, while violet laser 405 nm is the best laser to observe the red emission from both IR700 and porphyrin PICs. **(C).** By using Cary Eclipse UV-Vis-NIR spectroscopy, we showed the excitation of porphyrin at 488 nm would not interfere the flow cytometry study without using secondary antibody. The excitation by violet laser 405 nm cause red emission at 725 nm with 1.163 (a. u.) intensity, while the maximum red fluorescent intensity (3.779 a. u.) can be observed by excitation the Soret band of porphyrin at 432 nm.

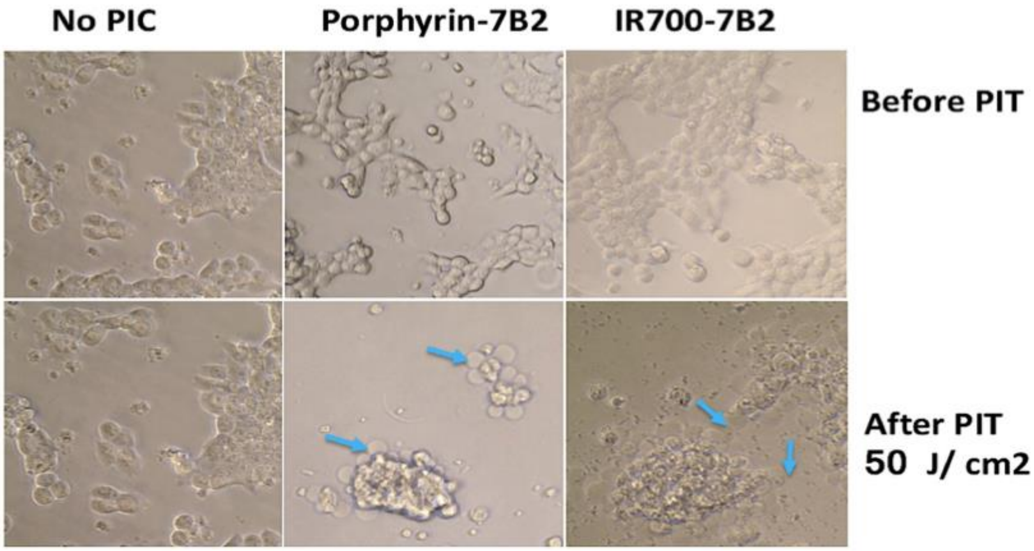

Supplemental Figure S2. Microscopic observations before and after PIT demonstrated the bleb formation (Blue arrows) as signs of necrotic cell death. Cells PIT-treated with IR700-7B2 showed not only rapid bleb formation but also cell debris. Control cells (No PIC) did not show any change during irradiation. Scale bars, 50 µm.

Supplementary Videos 1 and 2. Confocal microscopy videos of target-specific cell death in response to PIT in HIV Env-expressing 293T cells during 30 minutes irradiation. The cells were treated with IR700-7B2 or 7B2-porphyrin in the presence of sCD4 for 1 hour before laser irradiation by microscope.

Video 1: Red fluorescent emission from IR700-7B2 was directly detected. Rapid necrotic signs were observed in 5 minutes after irradiation of the adherent cells. After 30 minutes, cell debris were observed, as sign of necrotic cell death.

Video 2: As 7B2-porphyrin did not show strong red fluorescent emission, FITC anti-human IgG secondary antibody was applied to detect PIC indirectly. The cells were washed twice from unbound secondary antibodies, and suspended in the microscopic petri dish. Rapid internalization and necrotic signs were observed in 10 minutes after two-photon irradiation at 800 nm. Unlike IR700-antibody, no cell debris was observed after 30 minutes.

Supplementary Methods

Porphyrin-Antibody conjugation by click chemistry.

Conjugation experiment between azide porphyrin and mAb 7B2 was carried out in two steps: antibody functionalization, then clicked chemistry conjugation. The process is a modification of our protocol described previously (1,2). Briefly, methyl strained alkyne dibromopyridazinedione (MepStra PD) (15.0, μL, 20 mM in DMSO, 30 eq.) was added to a solution of 7B2 antibody (500 μL, 19 μM, 3.0 mg/mL) in borate buffer solution (BBS) (25 mM sodium borate, 25 mM NaCl, 0.5 mM EDTA, pH 8.0) and the solution was incubated at 4 °C for 1 hr. TCEP·HCl (6.0 μL, 20 mM in d.d water, 12 eq.) was added and the solution was incubated at 4 °C for a further 16 hr. Excess reagents were removed by ultrafiltration (6 × 3000 MWCO, VivaSpin¸ GE Healthcare) into BBS (pH = 8.0). Characterization was carried out on 50 μL of the resultant conjugate.

The protocol for synthesizing the water-soluble azide-porphyrin has been described elsewhere (3). Azide porphyrin (31 μL, 10 mM in DMSO, 40 eq.) was added to a solution of 7B2-rebridged conjugate (450 μL, 20 μM, 3.0 mg/mL) in BBS (25 mM sodium borate, 25 mM NaCl, 0.5 mM EDTA, pH 8.0) and the solution incubated at 21 °C for 4 hr. Excess reagents were removed by Zeba desalting column (Pierce) equilibrated with 1× PBS (pH = 7.4).

Conjugation and optimization of IR700-antibody by lysine modification.

The conjugation between IRDye 700DX and MAb 7B2 was done through an *N*-hydroxysuccinimide reactive group. To optimize the conjugation regarding the degree of labeling and photo-immuno efficacy, 7B2 antibody (800 μL, 7 μM, 1.1 mg/mL) in 1× PBS (pH = 8.5) was

mixed with varying equivalents of IRDye 700DX (50, 100 and 200 µg dye), equal to molar ratios of 3.5, 7 and 14, respectively. Finally, free dye was separated from labeled antibodies by Zeba desalting column (Pierce) equilibrated with PBS (pH = 7.4)., and the concentration of final products (7B2-IR700Dye) was measured by bicinchoninic acid (BCA) protein assay (Pierce, Rockford, IL).

UV-Vis-NIR Spectrophotometry

An Agilent Technologies Cary Series UV-VIS-NIR spectrofluorometer (Cary 5000) was used to observe the emission spectra of IR700-7B2 and porphyrin-7B2, excited at 689 nm and 432 nm, respectively. Observing the intensity of single excitation at 532 nm helped us for further study by DLS with incident light at 532 nm, as explained below.

Live imaging by two-photon confocal microscopy.

Live cell imaging was performed using a Zeiss LSM 780 confocal inverted microscope with a Coherent Chameleon laser (Ti:sapphire) as a source for two-photons (2P) excitation at 800 nm. The images were obtained by the average of 2 scans and no appreciate variation was observed. Live imaging was performed based on the time-series experiment consisting of image sequences with time intervals of 30s, and the nominal light dose delivered for each image pixel was 1 J/cm^2. The spatial resolution for 2P excitation was approximately 250 nm (considering the numerical aperture and the wavelength of excitation), as described previously (4).

One day before imaging, 10^4 Env-transfected 293T cells were seeded into 35 mm culture dishes with 0.17 mm thickness glass bottom (MatTek, Ashland, MA). In parallel, 293T cells were seeded as a control. Cells were cultured at 37°C in DMEM, 10% FCS, puromycin 1

µg/mL. The following day, cells were placed into 1 mL incomplete RPMI w/o Phenol red at pH 7.4 and transferred to the heated (37 °C) stages for confocal microscopy imaging. PICs and sCD4-183 were added to final concentrations of 1 µg/mL each. Taking images of different cells started after the addition of PICs; generally, 50 observations were acquired during the 30 min period of two-photon irradiation. Pinhole settings were such that an optical slice was less than 1 µm. After 30 min of imaging, the irradiated region was analyzed for live/dead by adding PI.

References

1. Bahou C, Richards DA, Maruani A, Love EA, Javaid F, Caddick S, et al. Highly homogeneous antibody modification through optimisation of the synthesis and conjugation of functionalised dibromopyridazinediones. Org Biomol Chem [Internet]. 2018;16(8):1359–66.

2. Castañeda L, Wright ZVF, Marculescu C, Tran TM, Chudasama V, Maruani A, et al. A mild synthesis of N-functionalised bromomaleimides, thiomaleimides and bromopyridazinediones. Tetrahedron Lett. 2013;54(27):3493–5.

3. Giuntini F, Bryden F, Daly R, Scanlan EM, Boyle RW. Huisgen-based conjugation of water-soluble porphyrins to deprotected sugars: towards mild strategies for the labelling of glycans. Org Biomol Chem [Internet]. 2014;12(8):1203–6.

4. Mello BL, Alessi AM, Riaño-Pachón DM, DeAzevedo ER, Guimarães FEG, Espirito Santo MC, et al. Targeted metatranscriptomics of compost-derived consortia reveals a GH11 exerting an unusual exo-1,4-β-xylanase activity. Biotechnol Biofuels. 2017;10(1):1–17.
